# Seroprevalence of SARS-CoV-2 Antibodies in Adults and Healthcare Workers in Southern Italy

**DOI:** 10.3390/ijerph18094761

**Published:** 2021-04-29

**Authors:** Francesco Napolitano, Gabriella Di Giuseppe, Maria Vittoria Montemurro, Anna Maria Molinari, Giovanna Donnarumma, Antonio Arnese, Maria Pavia, Italo Francesco Angelillo

**Affiliations:** 1 Department of Experimental Medicine, University of Campania “Luigi Vanvitelli”, Via L. Armanni, 5 80138 Naples, Italy; francesco.napolitano2@unicampania.it (F.N.); gabriella.digiuseppe@unicampania.it (G.D.G.); giovanna.donnarumma@unicampania.it (G.D.); antonio.arnese@unicampania.it (A.A.); maria.pavia@unicampania.it (M.P.); 2 Health Direction, Teaching Hospital of the University of Campania “Luigi Vanvitelli”, Via Santa Maria di Costantinopoli, 104 80138 Naples, Italy; mv.montemurro@libero.it; 3 Department of Precision Medicine, University of Campania “Luigi Vanvitelli”, Via L. De Crecchio, 7 80138 Naples, Italy; annamaria.molinari@unicampania.it

**Keywords:** adults, healthcare workers, Italy, SARS-CoV-2 antibodies, seroprevalence

## Abstract

Background: This study was carried out to estimate the seroprevalence of SARS-CoV-2 antibodies in a Southern Italian population. Methods: The study was performed among students and workers of the University of Campania “Luigi Vanvitelli” and the relative Teaching Hospital. Participants were invited to undergo a blood sampling, an interview or to complete a self-administered questionnaire. Results: A total of 140 participants (5.8%) tested positive for SARS-CoV-2 antibodies. Positive SARS-CoV-2 test results increased significantly during the months of testing, and those who had had at least one symptom among fever, cough, dyspnea, loss of taste or smell and who had had contact with a family member/cohabitant with confirmed COVID-19 were more likely to test positive. Faculty members were less likely to have a positive test result compared to the healthcare workers (HCWs). Among HCWs, physicians showed the lowest rate of seroconversion (5.2%) compared to nurses (8.9%) and other categories (10%). Nurses and other HCWs compared to the physicians, those who had had at least one symptom among fever, cough, dyspnea, loss of taste or smell, and who had had contact with a family member/cohabitant with confirmed COVID-19 were more likely to test positive. Conclusions: The results have demonstrated that SARS-CoV-2 infection is rapidly spreading even in Southern Italy and confirm the substantial role of seroprevalence studies for the assessment of SARS-CoV-2 infection circulation and potential for further spreading.

## 1. Introduction

The surveillance of the occurrence of COVID-19 cases is substantially based on the diagnostic tests using reverse transcriptase polymerase chain reaction (RT-PCR) that are provided to symptomatic patients, to contacts of COVID-19 cases, and, in certain circumstances, to asymptomatic subjects with specific characteristics, such as healthcare workers (HCWs). Since it has been shown that asymptomatic infections occur very frequently [1,2,3], and that these subjects and those pre-symptomatic can spread the infection [4,5,6], surveillance data on COVID-19 cases appear to be inadequate to picture the extent and to limit the spread of severe acute respiratory syndrome coronavirus 2 (SARS-CoV-2) circulation within populations. Moreover, comprehensive data on the burden of SARS-CoV-2 infection would be essential for the calculation of the infection fatality rate related to this novel coronavirus [7,8], and may also shed light on factors involved in the transmission among asymptomatic subjects.

Therefore, the availability of valid and reliable serologic tests for the detection of antibodies against SARS-CoV-2 has prompted the conduction of several epidemiological studies worldwide with the aim of estimating their prevalence in different populations [9,10,11,12], settings [13,14,15] and high-risk subjects, such as patients with underlying clinical conditions and HCWs [16,17,18]. 

In Italy, the initial course of the epidemic has determined an extraordinary and rapid development of the number of cases and deaths, characterized by a difference in the incidence between Northern and Southern regions, which suggested hypotheses involving demographic, geographic and genetic perspectives [19]. Instead, during the so called “second wave” of the pandemic, Southern regions have experienced an exponential increase in new COVID-19 cases starting from September 2020. To control the new massive spread of the SARS-CoV-2 infection, the Italian Government has promoted new restrictive measures, classifying the Regions into four areas (red, orange, yellow, white) according to the level of risk of infection. The measures for the containment of the epidemiological emergency include the limitation of travel and free circulation of persons, a night curfew, and the closure of urban spaces, sports facilities, schools and non-essential commercial activities according to the level of risk of infection periodically calculated in the Regions [20]. 

Despite these measures, the number of COVID-19 cases and deaths has increased dramatically and, to the best of our knowledge, up to date information on the proportion of subjects that have been infected and have produced antibodies against SARS-CoV-2 in Southern Italy is lacking [21,22,23,24,25], and it is reasonable to presume that a larger proportion of the population has been infected. 

Therefore, this study was carried out to estimate, by measuring the seroprevalence of SARS-CoV-2 antibodies, the extent of the circulation of SARS-CoV-2 in an adult population and in HCWs in Southern Italy and to evaluate which socio-demographic, anamnestic, and professional characteristics might predict the risk of infection by SARS-CoV-2 in these populations. 

## 2. Materials and Methods

### 2.1. Study Design, Population Recruitment and Procedures

The study was performed between 21 September 2020 and 31 December 2020, and it was part of a large project developed by the University of Campania “Luigi Vanvitelli” and the relative Teaching Hospital [26,27] in order to guarantee the safety of patients, HCWs, and the overall university community who accessed the healthcare and university facilities. The study population consisted of participants: (1) who were in contact with patients such as HCWs and medical students; (2) who were not in contact with patients, but with HCWs, such as technicians, laboratory assistants, custodians, cleaners, and administrative staff of the Teaching Hospital; (3) non-medical students, faculty members, research fellows and administrative staff of non-medical University Departments.

The data collection process has been described in previous research [26]. Briefly, students and workers who attended the University and Hospital facilities received an invitation by email to be voluntarily tested for antibodies against SARS-CoV-2, and those who responded were invited to the Health Surveillance ambulatory centers located in Caserta and Naples to perform a blood sampling and to undergo a structured interview or, if they preferred, to complete a self-administered questionnaire. 

In the waiting rooms of the ambulatory centers, the research team provided participants with the information about the study aims and the methods of data collection, and a signed consent form from each participant was obtained from those who were willing to participate. Prior to undergoing blood sampling, three well-trained investigators in data collection techniques invited the participants to undergo the interview or to complete the self-administered questionnaire. 

### 2.2. Survey Instrument

The questionnaire consisted of two sections. In the first one, the questions concerned participants’ socio-demographic (gender, age, marital status, education level), professional (professional role, whether they were HCWs, specific workplace and degree course (for students)), and anamnestic characteristics (weight and height, smoking status, presence and type of underlying clinical conditions, personal history of SARS-CoV-2 infection). In the second section, participants were asked whether they had been exposed to confirmed COVID-19 cases (cohabiting or non-cohabiting family members, friends, work or study colleagues, neighbors and patients), whether they had had COVID-19-compatible symptoms since February 2020 (headache, myalgia, fever, cough, dyspnea, tiredness, sore throat, nausea and vomiting, conjunctivitis, diarrhea, loss of taste or smell), whether they had been tested by RT-PCR for SARS-CoV-2 detection and the results of each testing, and the participants’ travel history outside Italy since February 2020.

### 2.3. Blood Sampling and Laboratory Methods

The blood samples were collected using test tubes with separator polymer gel (BD Vacutainer^®^ SST™ Tubes) and, after centrifugation, sera were stored at 4 °C until analysis. The detection of antibodies was performed within 24 h from sample collection in three laboratories located in the Teaching Hospital, using the following three chemiluminescence enzyme immunoassay (CLIA) tests: (1) total antibodies including IgM, IgG and IgA against SARS-CoV-2 using the VITROS ECiQ Immunodiagnostic Systems^®^ (Ortho-Clinical Diagnostics, Rochester, New York, NY, USA), an assay that employs luminol-horseradish peroxidase (HRP)-mediated chemiluminescence, with a sensitivity of 100% (95% CI = 92.7–100%) and a specificity of 100% (95% CI = 99.1–100%); samples with signal to cut-off (S/C) greater than or equal to 1 were considered positive; (2) detection of IgM and IgG using Abbott ARCHITECT i2000SR Instrument (Abbott Diagnostics, Chicago, IL, USA), with sensitivity for IgM and IgG of 95% and 100%, respectively, and specificity for IgM and IgG of 99.1% and 99.9%, respectively; samples with signal to cut-off (S/C) greater than or equal to 1.4 were considered positive; and (3) detection of IgM and S1/S2 IgG using LIAISON^®^ SARS-CoV-2 IgM qualitative test and S1/S2 IgG quantitative test (DiaSorin S.p.A., Saluggia, Italy), with a combined sensitivity of 98.3% (95% CI = 93.9–99.5%) and a specificity of 99.2% (95% CI = 98–99.7%); results above or equal to the 1.10 index indicated the presence of IgM antibodies against SARS-CoV-2 and samples with S1/S2 IgG >15.0 AU/mL were considered positive. All tests were performed according to the manufacturer’s instructions.

Subjects who tested positive for SARS-CoV-2 antibodies were invited to voluntarily undergo RT-PCR for SARS-CoV-2 detection from nasopharyngeal swabs.

### 2.4. Statistical Analysis

A descriptive analysis has been performed to describe the socio-demographic, professional and anamnestic characteristics of the participants overall and according to SARS-CoV-2 antibodies positivity. A bivariate analysis was carried out to evaluate the effect of the independent variables on the seropositivity for antibodies against SARS-CoV-2 in the overall sample and restricted to the group of HCWs using a chi-square test or Fisher’s exact test for the categorical variables and a Student’s t-test for the continuous variables. Then, a multivariate stepwise logistic regression analysis was performed to investigate the association of each independent variable with positivity for SARS-CoV-2 antibodies (Model 1), and the following variables were included: age (18–39 years = 1; 40–59 years = 2; ≥60 years = 3), gender (male = 0; female = 1), education level (high school degree or less = 0; college degree or higher = 1), marital status (unmarried/widowed/separated/divorced = 0; married/cohabiting = 1), population group (HCWs = 1; faculty members = 2; students = 3; research fellows = 4; administrative staff = 5; biologists/technicians = 6; other = 7), current smoking (no = 0; yes = 1), body mass index (BMI) (underweight/normal weight = 0; overweight/obese = 1), having at least one chronic medical condition (no = 0; yes = 1), travel history outside Italy in the previous ten months (no = 0; yes = 1), number of contacts with a confirmed COVID-19 case (none = 0; 1 = 1; 2 = 2; >2 = 3), contact with confirmed COVID-19 co-workers/study colleagues (no = 0; yes = 1), contact with confirmed COVID-19 family members/cohabitants (no = 0; yes = 1), having had at least one symptom among fever, cough, dyspnea, loss of taste or smell in the previous ten months (no = 0; yes = 1), and month of testing (September = 1; October = 2; November = 3; December = 4). The same model was performed after restriction to the HCWs group with the addition of the following variables: contact with confirmed COVID-19 patients (no = 0; yes = 1), professional role (physicians = 1; nurses = 2; other (nurse assistants, technicians, laboratory assistants)) = 3), working in wards where aerosol-producing procedures are performed (no = 0; yes = 1), and current working area (critical care/COVID-19 units = 1; medical = 2; surgical = 3; laboratory and diagnostics = 4) (Model 2).

Significance levels for exclusion and inclusion of variables in the models were *p*-values of 0.4 and 0.2, respectively. The results of the logistic regression analyses were reported as odds ratios (ORs) and 95% confidence intervals (CIs). All inferential tests were two-tailed with significant statistical levels for *p*-values equal to or less than 0.05. The statistical software Stata 15 [28] was used to carry out the analysis.

## 3. Results

A total of 2394 subjects voluntarily agreed to participate in the SARS-CoV-2 antibodies testing program. Table 1 displays the demographic, professional and anamnestic characteristics of the participants and the associated positivity for SARS-CoV-2 antibodies. More than one third (35.9%) were HCWs, 30.2% students, 17.3% administrative workers and 9.1% were faculty members. One in five reported to have at least one chronic disease (19.5%), and the most frequent were cardiovascular (30.8%), autoimmune (22.3%), allergies (21.6%) and respiratory diseases (15.4%), while 5.3% of the participants had diabetes. Almost one fifth (19.8%) had had contact with a confirmed COVID-19 case, 1.7% reported having contracted COVID-19, 515 (21.5%) had had COVID-19-compatible symptoms and 11.4% at least one symptom among fever, cough, dyspnea and loss of taste or smell from the beginning of the spread of the SARS-CoV-2 in Italy.

Overall, 140 participants (5.8%) tested positive for SARS-CoV-2 antibodies; specifically, 128 (84.2%) were positive for both IgM and IgG, 11 (7.9%) were IgM+IgG^−^, and 11 (7.9%) were IgM−IgG+, with a statistically significant time trend from September (2.9%) to December (8.7%) (χ^2^ = 11.41, *p* < 0.001). Of the 140 seropositive subjects, 98 (70%) voluntarily underwent nasopharyngeal swabs for RT-PCR SARS-CoV-2 detection, and four (4.1%) were diagnosed as COVID-19 cases.

Although not significantly, HCWs had the highest positive rate (7.1%), followed by biologists/technicians (6.6%), administrative staff (6.3%) and students (5.5%). Overall, among those who were not HCWs and non-medical students, 5.2% were positive to SARS-CoV-2 antibodies. Moreover, 26.4% of those who tested positive had had a close contact with confirmed COVID-19 cases, 26.4% were active smokers, and one in five (19.3%) had at least one chronic disease. At the bivariate analysis, the seroprevalence was significantly higher among participants who had had contacts with a confirmed COVID-19 case (7.8% vs. 5.4%; χ^2^ = 4.11, *p* = 0.04), and specifically with family members/cohabitants (22.2% vs. 5.4%; χ^2^ = 31.5, *p* < 0.001), those reporting COVID-19-compatible symptoms (9.5% vs. 4.9%; χ^2^ = 16.02, *p* < 0.001), or at least one symptom among fever, cough, dyspnea and loss of taste or smell (12.8% vs. 4.9%; χ^2^ = 26.95, *p* < 0.001) from the beginning of the spread of the SARS-CoV-2 infection. 

Most of these results were confirmed after adjustment through the multivariate logistic regression analysis, that showed that positive SARS-CoV-2 tests increased significantly during the months of testing (OR = 1.4; 95% CI = 1.13–1.74). Moreover, participants who had had at least one symptom among fever, cough, dyspnea, loss of taste or smell in the previous ten months (OR = 2.98; 95% CI = 1.94–4.56) and those who had had contact with a family member/cohabitant with confirmed COVID-19 (OR = 8.58; 95% CI = 2.14–34.34) were more likely to test positive for SARS-CoV-2 antibodies. Instead, faculty members were less likely to have a positive test result compared to the HCWs (OR = 0.3; 95% CI = 0.12–0.76) (Model 1 in Table 2). The significant association between having had at least one symptom among fever, cough, dyspnea, loss of taste or smell and the positivity to SARS-CoV-2 antibodies persisted also after the exclusion from the analysis of participants with a COVID-19 diagnosis before the study (OR = 1.83; 95% CI = 1.08–3.1) (data not shown).

Table 3 reports the descriptive and univariate analysis restricted to HCWs. Within this subgroup, which, as mentioned, showed the highest seroprevalence of SARS-CoV-2 antibodies, physicians were the professional category that showed the lowest rate of seroconversion (5.2%), compared to nurses (8.9%) and other categories of HCWs (10%), and these differences almost achieved statistical significance (χ^2^ = 5.95, *p* = 0.051). Seroprevalence also differed, although not significantly, according to hospital area, ranging from 5.9% in HCWs attending the medical wards to 8.3% in those working in the critical care/COVID-19 units, and even for HCWs, contacts with COVID-19 family members/cohabitants were significantly associated to positivity to SARS-CoV-2 antibodies. Of the 40 reported COVID-19 cases, 33 (82.5%) were HCWs; specifically, 15 (45.4%) were physicians, 12 (26.4%) nurses and 6 (18.2%) other HCWs (nurse assistants, technicians, laboratory assistants). For the other tested characteristics, compared to the overall population, no relevant differences were found at the univariate analysis. 

In the logistic regression model investigating associations with positivity to SARS-CoV-2 antibodies in HCWs the results confirmed that nurses (OR = 2.1; 95% CI = 1.07–4.13) and other HCWs, including nurse assistants, technicians and laboratory assistants (OR = 2.57; 95% CI = 1.29–5.14), compared to the physicians, had a significantly higher probability of testing positive for SARS-CoV-2 antibodies, as well as those who had had at least one symptom among fever, cough, dyspnea, loss of taste or smell (OR = 4.47; 95% CI = 2.25–8.89), and those who had had contact with a family member/cohabitant with confirmed COVID-19 (OR = 8.5; 95% CI = 1.74–41.5) (Model 2 in Table 2). When HCWs with a COVID-19 diagnosis before study were excluded from the logistic regression analysis, having had at least one symptom among fever, cough, dyspnea, loss of taste or smell was no more significantly associated to positivity to SARS-CoV-2 antibodies (data not shown).

## 4. Discussion

The present study reports the results of a comprehensive project that has investigated the circulation of the SARS-CoV-2 infection through the assessment of the seroprevalence of antibodies in a university population in Southern Italy. This is, to our knowledge, the first study analyzing the spread of the SARS-CoV-2 infection during the “second wave” of the pandemic, that has affected the southern regions of the country with a relevantly higher burden of cases and deaths compared to the first one. 

The main finding of the study is that, in the period from September to December 2020, an overall anti-SARS-CoV-2 antibodies seroprevalence of 5.8% was revealed in the investigated population, and that this circulation was time-dependent, with a remarkable trend ranging from 2.9% in September to 8.7% in December. These results stimulate a series of considerations on the course of the pandemic in this area and on the role of seroprevalence studies that deserve to be mentioned. First of all, they suggest that only the very early implementation of stringent public health control measures in Southern Italy, including the strict lockdown during the so called “first wave”, that were established when the circulation of SARS-CoV-2 was still very low in the area, were able to contain the diffusion in Southern Italy, as revealed by a national seroprevalence study that showed a value less than 1% in Southern regions [29], whereas the milder measures implemented following the summer months were not so effective in the control of the SARS-CoV-2 spread during the “second wave”, with an almost tripled prevalence in less than four months. An even faster spread has been reported in Switzerland over the course of a five-week study, with an increase in seroprevalence from 5% to 11% [11]. Moreover, the results suggest, consistent with previous studies [9,10,24], that data on seroprevalence reflect a more realistic picture of the spread of the infection, that, also in this context, goes far beyond the results showed by the surveillance of confirmed COVID-19 cases, demonstrating the potentials for SARS-CoV-2 transmission through asymptomatic individuals. 

Since previous seroprevalence studies differ, for example, in the involved populations, sample selection strategies and chosen laboratory tests, the comparability of results is hard to obtain, and the differences might be more related to study design and methodologies than to a variable SARS-CoV-2 circulation in the involved populations. Indeed, numerous studies have been conducted worldwide, and wide differences have been reported (0.9–35.1%) [8,10,11,14,25,30]. The results of two previous investigations conducted in a large geographic area of Northern Italy showed, as expected, higher rates of seropositivity, since 23% of blood donors [24] and 11% of non-hospitalized participants [25] had antibodies against SARS-CoV-2; instead, the seroprevalence was 0.99% in a sample of blood donors in a southern region [31]. Interestingly, in this investigation, 5.5% of university students were positive for SARS-CoV-2 antibodies, and this rate is comparable with the result of 4% in a study conducted in the US on college students [32], whereas it was higher than that observed in Greek students (0.72%) [15] and in Spain among a sample of students, faculty and administrative staff (2.89%) [33]. Instead, higher seroprevalence rates were observed in Chile among students (9.9%) and staff (16.6%) [34] and in the US among campus students (31.2%) [35]. No differences in SARS-CoV-2 seroprevalence were found according to several demographic characteristics, and this has already been reported in the literature [9,30,36]. Analogously, obesity and chronic diseases were not predictors of positivity to SARS-CoV-2 antibodies. However, many investigations have demonstrated that these conditions are associated with a high risk of severe complications and death from COVID-19 [37,38,39,40] and of symptomatic COVID-19, although the association to a higher susceptibility to SARS-CoV2 is still controversial [41,42,43,44,45]. Further studies on the role of these conditions on the susceptibility to SARS-CoV-2 in asymptomatic subjects are warranted. The finding that close contacts with people with COVID-19, particularly those in the same household, is associated with increased odds of seroconversion, even in subjects that were not aware of having been infected, suggests that there has been a relevant number of individuals that were eligible for RT-PCR testing but have not undergone it. Missed opportunities for RT-PCR testing were also revealed by the finding that positivity to SARS-CoV-2 was significantly higher in those reporting COVID-19-compatible symptoms, showing that even symptomatic or pauci-symptomatic individuals did not receive diagnostic tests. The role of COVID-19-compatible symptoms as predictors of seropositivity has also been reported in other studies conducted in Italy, Europe and US [8,9,25,32,46] and suggests that the surveillance of COVID-19 cases underestimated even the occurrence of symptomatic cases. It should also be remarked that the definition of the “asymptomatic” has been reported to be challenging and prone to limitations, since it is based on self-reported clinical symptoms and is evolving and conditioned by subjectivity [47].

As expected, the prevalence of SARS-CoV-2 antibodies was higher in HCWs (7.1%) compared to all other investigated subgroups, and it is also worth noting that more than 80% of the reported COVID-19 cases were HCWs, confirming the occupational risk for both asymptomatic and symptomatic infections. This occurred even though the Teaching Hospital implemented all the recommended measures to limit the spread of the SARS-CoV-2 infection (mandatory use of face masks, hand washing, distancing, etc.) and limited the access of visitors and caregivers. Furthermore, non-urgent surgical procedures have been postponed, and this has resulted in reductions in terms of surgical volume, diagnoses and hospitalizations [48,49,50,51]. Large differences in seroprevalence have been reported among HCWs, but the comparisons are undermined by the difficulty to distinguish the role of occupational risks to that related to the underlying SARS-CoV-2 infection community prevalence [52], and this seems to be confirmed by the finding, detected in the present and in previous studies [53,54], that even in the subgroup of HCWs, SARS-CoV-2 antibodies seropositivity is strongly associated to contact with COVID-19 family members/cohabiting rather than with patients and workplace colleagues. The rate of positivity among HCWs found in this study was higher compared to the results of other investigations conducted in Italy among HCWs in hospital settings [23,55], and, interestingly, also compared to that reported in a study conducted in the same area among HCWs working with suspected and confirmed cases of COVID-19 (3.5%) [22] and in a study conducted in Tuscany (4.1%) [56]—although during the first wave of the pandemic, when, as expected, it was lower compared to the findings in the geographic areas of Brescia [54] and Milan [57] (Lombardy region), where the prevalence of antibodies against SARS-CoV-2 were 8.6% and 14.3%, respectively. 

Within HCWs, physicians showed the lowest seroprevalence, compared to nurses and other HCWs, and this finding is consistent with previous studies conducted in Sweden [18] and Italy [55], where nurses and healthcare assistants were more likely to test positive. Moreover, no significant differences were revealed according to working area, and this result is more controversial, with studies confirming this finding [52,58] and others that found significant associations between working in COVID-19 wards or having had contact with patients with COVID-19 and HCWs’ seropositivity [18,59]. Taken together, the results of the present study suggest that the occupational risk might be more related to the specific professional practice rather than to the characteristics of the treated patients and to the workplace. Finally, in contrast with our finding in the overall population, no association with the occurrence COVID-19-compatible symptoms was found with SARS-CoV-2 antibodies seropositivity, suggesting stricter diagnostic protocols in symptomatic HCWs as compared to the general population.

The results of this survey should be analyzed bearing in mind that the mechanisms of the immune response to SARS-CoV-2 infection are still unknown in many aspects. Although it has been demonstrated that seroconversion occurs also in asymptomatic subjects [1,3], debate still exists regarding the duration of detectable antibody titers in both symptomatic and asymptomatic individuals [60,61] and whether this persistence is related to the severity of the disease and/or specific characteristics of subjects (comorbidities, age, etc.) [62,63], as well as regarding the protection against upcoming SARS-CoV-2 infections [64]. Despite all these uncertainties, seroprevalence studies represent a very powerful instrument to have an insight in the cumulative spread of SARS-CoV-2 within populations, and in our specific context it has demonstrated that the extent of the circulation has begun to be relevant by the end of summer and has steadily increased through the end of the year. These results provide evidence of the usefulness of repeated seroprevalence surveys that, for the future, should also take into account the effects of the COVID-19 mass vaccination campaign launched in Europe and Italy on 27 December 2020, for the implications they will have on the assessment of the burden of past SARS-CoV-2 infections and the potential for their further spread in the community, particularly the asymptomatic cases or those symptomatic that have been missed by the surveillance based on laboratory confirmed COVID-19 cases, and for monitoring the community coverage for the achievement of the herd immunity threshold. Indeed, recent evidence suggests that extensive immunization against SARS-CoV-2 could slow the spread of the infection [65,66]. 

Several limitations should be acknowledged in the interpretations of the results of this study. A convenience voluntary sample was recruited, and considerations on external validity are warranted, since the effect of the willingness to participate and the representativeness of the recruited population on seroprevalence estimates are difficult to establish. However, the peculiar setting that was investigated might allow us to consider the seroprevalence as representative of the entire population of HCWs in our area, and the remaining sample as representative of the adults in Southern Italy. Nevertheless, confirmation of these seroprevalence estimates through a probabilistic representative sample would be useful. Moreover, serologic tests are subjected to errors with false-positive or false-negative results, with the false positives being of more concern in populations with an expected low seroprevalence. However, the declared sensitivity and specificity of the involved tests were very high, and we may be confident that the false positives and negatives were not numerous enough to have a relevant influence on the final seroprevalence estimates. Furthermore, the data on seroprevalence relied on the use of different types of tests, that might have influenced the results; nonetheless, the performances of the tests are claimed to be similar to each other [67,68,69]. Finally, the cross-sectional nature of the study with the simultaneous assessment of exposures and outcomes provides no evidence of temporal relationships among variables of interest, and the retrospective assessment of self-reported symptoms, as well as of backdated exposures, may be subjected to misclassification.

In conclusion, the results of the study have demonstrated that the SARS-CoV-2 infection is rapidly spreading even in Southern Italy and far beyond the data revealed by COVID-19 cases surveillance, and confirm the substantial role of seroprevalence studies for the assessment of SARS-CoV-2 infection circulation and the potential for further spreading. Repeated seroprevalence surveys are warranted coupled with the evaluation of the effectiveness of the COVID-19 mass vaccination strategy.

## Figures and Tables

**Table 1:** Demographic, professional and anamnestic characteristics of the participants and the associated positivity to SARS-CoV-2 antibodies.

Characteristic	Overall Population *n* = 2394		SARS-CoV-2 Antibody Positive *n* = 140	
	*n*	*%*	*n*	*%*
Gender				
Female	1423	59.4	83	5.9
Male	971	40.6	57	5.8
			χ^2^ = 0.001; *p* = 0.969	
Age, years				
18–39	1446	60.4	89	6.1
40–59	735	30.7	42	5.7
≥60	213	8.9	9	4.2
			χ^2^ = 1.29; *p* = 0.525	
Education level				
College degree or higher	1486	62.1	88	5.9
High school degree or less	908	37.9	52	5.7
			χ^2^ = 0.039; *p* = 0.844	
Marital status				
Unmarried/widowed/separated/divorced	1484	62	89	6
Married/cohabiting	910	38	51	5.6
			χ^2^ = 0.158; *p* = 0.691	
BMI				
Overweight/obese	860	35.9	54	6.3
Under/normal weight	1534	64.1	86	5.7
			χ^2^ = 0.453; *p* = 0.501	
Current smoking				
Yes	582	24.3	37	6.4
No	1812	75.7	103	5.7
			χ^2^ = 0.362; *p* = 0.547	
Having at least one chronic medical condition				
Yes	468	19.5	27	5.8
No	1926	80.5	113	5.9
			χ^2^ = 0.006; *p* = 0.963	
Population group				
HCWs	859	35.9	61	7.1
Biologists/Technicians	76	3.2	5	6.6
Administrative staff	415	17.3	26	6.3
Students	723	30.2	40	5.5
Other	67	2.8	2	3
Research fellows	36	1.5	1	2.8
Faculty members	218	9.1	5	2.3
			Fisher’s exact *p* = 0.137	
Travel history outside Italy in the previous 10 months				
Yes	190	7.9	16	8.4
No	2204	92.1	124	5.6
			χ^2^ = 2.48; *p* = 0.115	
COVID-19 diagnosis before study				
Yes	40	1.7	30	75
No	2354	98.3	110	4.7
			χ^2^ = 353.3; *p* < 0.001	
Contact with a confirmed COVID-19 case				
Yes	474	19.8	37	7.8
No	1920	80.2	103	5.4
			χ^2^ = 4.11; *p* = 0.04	
Number of contacts with a confirmed COVID-19 case ^				
>2	51	10.8	5	9.8
2	95	20	9	9.5
1	328	69.2	23	7
			Fisher’s exact *p* = 0.589	
Contact(s) with confirmed COVID-19 co-workers/study colleagues				
Yes	368	15.4	24	6.5
No	2026	84.6	13	5.7
			χ^2^ = 0.34; *p* = 0.559	
Contact(s) with confirmed COVID-19 family members/cohabitants				
Yes	63	2.6	14	22.2
No	2331	97.4	126	5.4
			χ^2^ = 31.5; *p <* 0.001	
Having had at least one COVID-19-compatible symptom in the previous ten months				
Yes	515	21.5	49	9.5
No	1879	78.5	91	4.9
			χ^2^ = 16.02; *p* < 0.001	
Having had at least one symptom among fever, cough, dyspnea, loss of taste or smell in the previous ten months				
Yes	274	11.4	35	12.8
No	2120	88.6	105	4.9
			χ^2^ = 26.95; *p* < 0.001	
Having undergone at least one screening test with RT-PCR for SARS-CoV-2 detection in the previous ten months				
Yes	1111	46.4	71	6.4
No	1283	53.6	69	5.4
			χ^2^ = 1.108; *p* = 0.292	
Month of testing				
December	127	5.3	11	8.7
November	752	31.4	56	7.5
October	1110	46.4	61	5.5
September	405	16.9	12	2.9
			χ^2^ trend = 11.41; *p* < 0.001	

^ Among those who had had contact with a confirmed COVID-19 case.

**Table 2:** Results of multivariate logistic regression analysis investigating the factors associated with positivity to SARS-CoV-2 antibodies.

Variable	OR	SE	95% CI	*p*
Model 1. Positivity to SARS-CoV-2 antibodies (Sample size = 2394)				
Log likelihood = −499.85, χ^2^ = 66.88(14 df), *p* < 0.0001				
Having had at least one symptom among fever, cough, dyspnea, loss of taste or smell in the previous ten months	2.98	0.65	1.94–4.56	<0.001
Contact(s) with confirmed COVID-19 family members/cohabitants	8.58	6.07	2.14–34.34	0.002
Month of testing (September through December 2020)	1.4	0.15	1.13–1.74	0.002
Population group				
HCWs	1 *			
Faculty member	0.3	0.14	0.12–0.76	0.011
Students	0.66	0.14	0.43–1.01	0.051
Research fellows	0.36	0.37	0.05–2.75	0.327
Other	0.3	0.22	0.07–1.29	0.107
Administrative staff	Backward elimination			
Technicians/Biologists	Backward elimination			
Travel history outside Italy in the previous ten months	1.69	0.49	0.96–2.98	0.067
Number of contacts with a confirmed COVID-19 case				
None	1 *			
1	0.33	0.23	0.09–1.26	0.107
2	0.42	0.33	0.09–1.94	0.266
>2	0.29	0.28	0.04–1.99	0.209
Contact(s) with confirmed COVID-19 co-workers/study colleagues	2.45	1.71	0.63–9.53	0.196
Age				
18–39 years	1 *			
>59 years	0.64	0.24	0.31–1.32	0.233
40–59 years	Backward elimination			
BMI				
Under/normal weight	1 *			
Overweight/obese	1.23	0.23	0.85–1.77	0.264
Model 2. Positivity for SARS-CoV-2 antibodies among HCWs (Sample size = 859)				
Log likelihood = −194.17, χ^2^ = 51.89(12 df), *p* < 0.0001				
Having had at least one symptom among fever, cough, dyspnea, loss of taste or smell in the previous ten months	4.47	1.57	2.25–8.89	<0.001
Month of testing (September through December 2020)	1.65	0.27	1.19–2.28	0.003
Professional role				
Physicians	1 *			
Nurses	2.1	0.73	1.07–4.13	0.032
Other (nurse assistants, technicians, laboratory assistants)	2.57	0.9	1.29–5.14	0.007
Contact(s) with confirmed COVID-19 family members/cohabitants	8.5	6.87	1.74–41.5	0.008
Age				
18–39 years	1 *			
40–59 years	0.56	0.19	0.28–1.09	0.086
>59 years	0.59	0.28	0.23–1.51	0.276
Male HCWs	0.63	0.18	0.36–1.11	0.109
Number of contacts with a confirmed COVID-19 case				
None	1 *			
1	0.37	0.29	0.08–1.71	0.205
2	0.45	0.41	0.07–2.72	0.385
>2	0.38	0.4	0.05–3.05	0.365
Contact(s) with confirmed COVID-19 co-workers	2.18	1.72	0.46–10.22	0.325

* Reference category. The following variables were removed from the models by the backward elimination procedure: gender, marital status, education level, current smoker and having at least one chronic medical condition (Model 1); marital status, education level, BMI, having at least one chronic medical condition, current working area, working in wards where aerosol-producing procedures are performed and travel history outside Italy in the previous ten months (Model 2).

**Table 3:** Demographic, professional and anamnestic characteristics of the HCWs and the associated positivity to SARS-CoV-2 antibodies.

Characteristic	Overall Population *n* = 859		SARS-CoV-2 Antibody Positive *n* = 61	
	*n*	*%*	*n*	*%*
Gender				
Male	367	42.7	31	8.4
Female	492	57.3	30	6.1
			χ^2^ = 1.76; *p* = 0.185	
Age, years				
18–39	508	59.1	40	7.9
40–59	249	29	15	6
≥60	102	11.9	6	5.9
			χ^2^ = 1.13; *p* = 0.569	
Education level				
High school degree or less	101	11.8	11	10.9
College degree or higher	758	88.2	50	6.6
			χ^2^ = 2.49; *p* = 0.117	
Marital status				
Unmarried/widowed/separated/divorced	460	53.5	33	7.2
Married/cohabiting	399	46.5	28	7
			χ^2^ = 0.16; *p* = 0.691	
BMI				
Overweight/obese	309	36	24	7.8
Under/normal weight	550	64	37	6.7
			χ^2^ = 2.22; *p* = 0.329	
Current smoking				
Yes	241	28.1	14	5.8
No	618	71.9	47	7.6
			χ^2^ = 0.36; *p* = 0.547	
Professional role				
Others (nurse assistants, technicians, laboratory assistants)	170	19.8	17	10
Nurses	224	26.1	20	8.9
Physicians	465	54.1	24	5.2
			χ^2^ = 5.95; *p* = 0.051	
Current working area				
Critical care/COVID-19 units	108	12.6	9	8.3
Surgical	260	30.3	21	8.1
Laboratory and Diagnostics	121	14.1	9	7.4
Medical	370	43.1	22	5.9
			χ^2^ = 1.39; *p* = 0.707	
Having at least one chronic medical condition				
Yes	175	20.4	13	7.4
No	684	79.6	48	7
			χ^2^ = 0.03; *p* = 0.85	
Travel history outside Italy in the previous ten months				
Yes	48	5.6	4	8.3
No	811	94.4	57	7
			Fisher’s exact *p* = 0.769	
COVID-19 diagnosis before study				
Yes	33	3.8	24	72.7
No	826	96.2	37	4.5
			χ^2^ = 224.1; *p* < 0.001	
Contact with a confirmed COVID-19 case				
Yes	331	38.5	27	8.2
No	528	61.5	34	6.5
			χ^2^ = 0.91; *p* = 0.340	
Number of contacts with a confirmed COVID-19 case ^				
>2	39	11.8	4	10.3
2	70	21.1	6	8.6
1	222	67.1	17	7.7
			Fisher’s exact *p* = 0.749	
Contact(s) with confirmed COVID-19 co-workers				
Yes	252	29.3	17	6.7
No	607	70.7	44	7.2
			χ^2^ = 0.06; *p* = 0.794	
Contact(s) with confirmed COVID-19 patients				
Yes	69	7.8	3	4.4
No	790	92.2	58	7.3
			Fisher’s exact *p* = 0.468	
Contact(s) with confirmed COVID-19 family members/cohabitants				
Yes	35	4.1	10	28.6
No	824	95.9	51	6.2
			Fisher’s exact *p* < 0.001	
Having had at least one COVID-19-compatible symptom in the previous ten months				
Yes	188	21.9	23	12.2
No	671	78.1	38	5.7
			χ^2^ = 9.61; *p* = 0.002	
Having had at least one symptom among fever, cough, dyspnea, loss of taste or smell in the previous ten months				
Yes	78	9.8	16	20.8
No	781	90.2	45	5.7
			χ^2^ = 23.4; *p* < 0.001	
Having undergone at least one screening test with RT-PCR for SARS-CoV-2 detection in the previous ten months				
Yes	782	91.1	49	6.3
No	77	8.9	12	15.6
			χ^2^ = 9.23; *p* = 0.002	
Month of testing				
December	94	10.9	10	10.6
November	389	41.8	32	8.9
October	229	26.7	14	6.1
September	177	20.6	5	2.8
			χ^2^ trend = 8.64; *p* = 0.003	

^ Among those who had had contact with a confirmed COVID-19 case.

## Data Availability

The data presented in this study are available on request from the corresponding author.
